# Automated Recognition of Nanoparticles in Electron Microscopy Images of Nanoscale Palladium Catalysts

**DOI:** 10.3390/nano12213914

**Published:** 2022-11-06

**Authors:** Daniil A. Boiko, Valentina V. Sulimova, Mikhail Yu. Kurbakov, Andrei V. Kopylov, Oleg S. Seredin, Vera A. Cherepanova, Evgeniy O. Pentsak, Valentine P. Ananikov

**Affiliations:** 1 Zelinsky Institute of Organic Chemistry, Russian Academy of Sciences, 119991 Moscow, Russia; 2 Tula State University, Lenine Ave. 92, 300012 Tula, Russia

**Keywords:** nanoscale catalysts, computational analysis, machine learning, supported catalysts, palladium nanoparticles

## Abstract

Automated computational analysis of nanoparticles is the key approach urgently required to achieve further progress in catalysis, the development of new nanoscale materials, and applications. Analysis of nanoscale objects on the surface relies heavily on scanning electron microscopy (SEM) as the experimental analytic method, allowing direct observation of nanoscale structures and morphology. One of the important examples of such objects is palladium on carbon catalysts, allowing access to various chemical reactions in laboratories and industry. SEM images of Pd/C catalysts show a large number of nanoparticles that are usually analyzed manually. Manual analysis of a statistically significant number of nanoparticles is a tedious and highly time-consuming task that is impossible to perform in a reasonable amount of time for practically needed large amounts of samples. This work provides a comprehensive comparison of various computer vision methods for the detection of metal nanoparticles. In addition, multiple new types of data representations were developed, and their applicability in practice was assessed.

## 1. Introduction

Currently, heterogeneous catalysis is one of the most important fields of chemistry due to its high applicability in industrial processes [1]. The ease of catalyst recovery and high turnover numbers make heterogeneous catalysts useful for large-scale production. One of the most critical commercial catalysts is palladium on carbon (Pd/C). As a few examples, it can catalyze various reactions, including [2] but not limited to hydrogenations [3], hydrogenolysis [4], cross-coupling [5,6], and others [7]. The highest catalyst performance with ~10^9^ turnover numbers was recently achieved with Pd/C catalysts [8]. Representative applications also include sensing [9,10], hybrid nanomaterials design [11,12,13,14], development of Pd-decorated nanosystems on various forms of carbon [15,16,17], and biosynthesis [18], among many others.

Undoubtedly, the optimization of catalytic performance is an important task. The activity of the catalyst is strongly related to its structure and its surface structure in particular. Therefore, the description of the surface becomes a key task; one of the possible criteria used for this is the imperfection of the surface, i.e., the presence of defects. Defects are interruptions of the regular structure of the carbon surface. The most versatile method of material morphology analysis is scanning electron microscopy [19]. The sample is scanned with a beam of electrons, and backscattered electrons are then detected. However, even with this method, some types of defects are poorly visualized due to the slight difference in the atomic arrangement. One of the approaches for visualizing defects is the deposition of metal particles. Due to the preferential binding of particles to defects [19,20], the formed patterns correspond with sufficient accuracy to the distribution of defects; and using the Z-contrast associated with atomic number, the electron microscopy accurately determines the location of the particles. Since scanning microscopy is a nondestructive method, hundreds of thousands of images can be produced for each sample, which in turn makes manual analysis impossible [21]. While methods for automatic imaging of the sample exist, their results are tedious to interpret.

To address these issues, computer vision solutions were developed. The list of approaches can be separated into two large groups: classical computer vision techniques and deep learning methods. The first ones are based on either some sort of binarization technique or by fitting a function to match the brightness profile of the image. The second ones include direct detection techniques (R-CNN, faster R-CNN, mask R-CNN) or detection via segmentation approaches (for instance, segmentation first [22] and then applying the Watershed algorithm). As a few representative examples, computer vision is a highly promising technique for connecting microscopy with nanoscale materials and catalysts [23,24], for performing mechanical analysis [25], and carrying out challenging nanoscale multi-manipulations in scanning electron microscopy (SEM) [26].

One of the most challenging problems in processing SEM images is the construction of an appropriate dataset. Examples of SEM image datasets include the dataset of a wide range of objects studied with SEM [27] and the synthetic dataset of powder materials [28]. Most available datasets include only image labels, as labeling images for segmentation or detection is enormously time-consuming. The dataset with SEM images of Pd/C catalysts [29] was used in this work.

This work presents an in-depth comparison of classical and deep learning approaches. We show that high quality can be achieved without using complex neural methods. The developed pipeline was used to analyze images of Pd/C catalysts and helped draw insightful conclusions about the underlying material structure.

## 2. Results and Discussion

### 2.1. General Approach

The experimental setup included four manually labeled SEM images of Pd/C catalysts. The labeling was performed by an expert proficient in analyzing such images. For every nanoparticle, its center and radius were obtained.

These images were used to compare the performance of six different object-detection methods, choose the most accurate of them in terms of the accepted measure of nanoparticle detection accuracy, and adjust its parameters.

The best method was then used to make predictions for the entire dataset of SEM Pd/C images [30]. This made it possible to infer unique particle-distribution patterns depending on the sample, i.e., the type of carbon material used for the catalyst preparation. These data may be crucial for constructing relationships between catalytic activity and support properties.

### 2.2. Methods Comparison

In this section, the performance of six different object-detection methods is compared:

- The straightforward local intensity maxima (LM) method;
- Three classical computer vision methods of blob detection: Laplacian of Gaussian (LOG), difference of Gaussians (DOG), and determinant of the Hessian (DOH) methods;
- The proposed methods in this work include exponential approximation method (Exp) and
- Deep neural network (DNN) approach.

A more detailed description of the methods is presented below in the “Method Implementation” section.

To estimate the possibilities of algorithms to detect nanoparticles on SEM images, we tested their performance for each of the four labeled ground truth images for different parameter values. Table 1 contains accuracy values (in %) for the optimal parameter values obtained from the parameter search. The last column of Table 1 contains the mean values and standard deviations of accuracy computed through four images.

All the results were obtained for the object acceptability threshold equal to 0.25 according to the Jaccard measure (1). Note that the rectangular bounding box for representing the position and size of an object, which is typical for computer vision, is unnatural for detecting nanoparticles due to their spherical shape, which leads to the overestimation of the Jaccard measure. Therefore, we use a circular region of interest here. A detailed description can be found in the Section 4.2 “Metric Calculation”.

**Figure 1:**
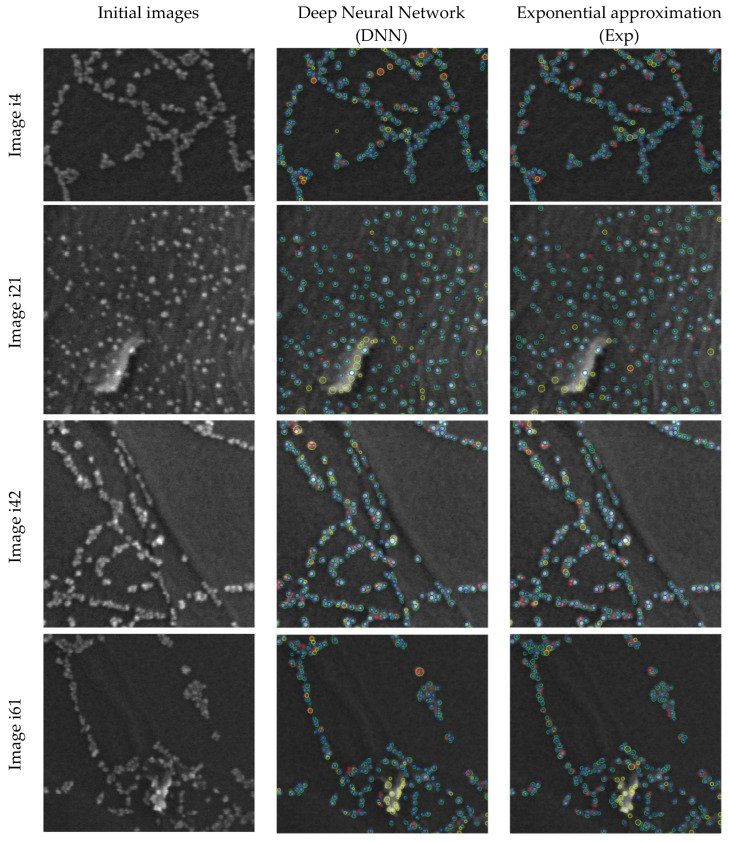
Visualization of the results of nanoparticle detection for the DNN and Exp methods.

Figure 1 presents initial images and the visualization of the results of nanoparticle detection for two methods (DNN and Exp) that show the best quality on average for its optimal parameter values. In this figure, particles found by the algorithm are marked in green, those manually labeled are in blue, particles found by the algorithm but not confirmed by the expert labeling are in yellow, and particles marked by the expert but not found by the algorithm are in red.

To model the real situation, when we do not have any expert information for image analysis, we chose algorithm parameters based on the leave-one-out procedure [31]. Namely, four times in a turn, we selected one of the four images as the test image, found the set of parameters that gives the best sum of accuracies for the three remaining images, and applied this set to process the test image. Thus, the average accuracy for four test images provides the final estimation of the algorithm performance. Such a procedure, while time-consuming, is recognized as valid in data analysis, especially in cases with limited amounts of data.

The results of such a leave-one-out procedure are presented in Table 2. The four central columns of this table contain the obtained accuracy (in %) for the specified test images, and the last column contains the average accuracy values and the corresponding standard deviations calculated from all four test images.

As we can see from Table 2, the accuracies of nanoparticle detection for parameter estimation based on the leave-one-out procedure, as expected, are lower than the respective values for optimal parameter values. This means that the methods are sensitive to the choice of parameters, and the detection accuracy can be further improved by introducing a more suitable way of estimating the parameters. 

According to Table 2, the most accurate method for the adopted experimental setup is an exponential approximation. Additionally, it is most stable towards changes of parameter values. Therefore, it was selected for further study.

### 2.3. Analysis of the Data

The exponential approximation method (Exp), which showed the best quality on the labeled data, was applied to analyze the entire dataset of SEM Pd/C images [30].

The main parameters of the Exp method are stated as follows:

- Size of image fragments for approximation fsize=7,
- Set of possible radius values: from 1 to 7 through 0.1,
- Values of adaptive threshold coefficients for prefiltering $Cf_{pref}$ (stage 2) and detection ${Cf}_{detect}$ (stage 4) were estimated as parameters that maximize the sums of accuracies for all four manually labeled images: $Cf_{pref}=0.3$, $Cf_{detect}=0.4$.

As a result of applying the Exp method to each dataset image, the set of nanoparticles was obtained, each of which is characterized by the coordinates of its center and radius.

**Figure 2:**
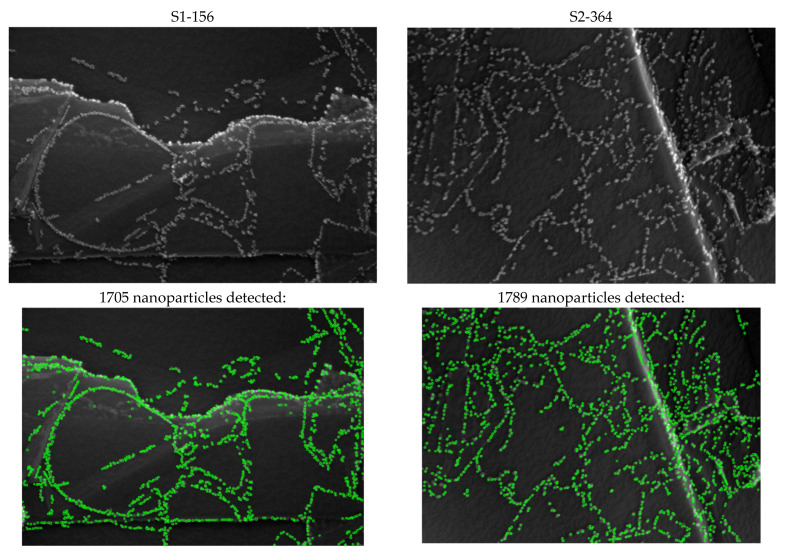
Examples of images of types S1 (**left**) and S2 (**right**) from the base and the results of nanoparticle detection.

Based on this information, the number of detected nanoparticles was found for each image, and a number of statistical data were calculated to characterize images to distinguish between two kinds of them, named S1 and S2. Figure 2 shows an example of two representative S1 and S2 images from the base and the results of nanoparticle detection.

Figure 3 shows the main obtained statistical data visual presentation for images S1-156 and S2-364 from Figure 2.

**Figure 3:**
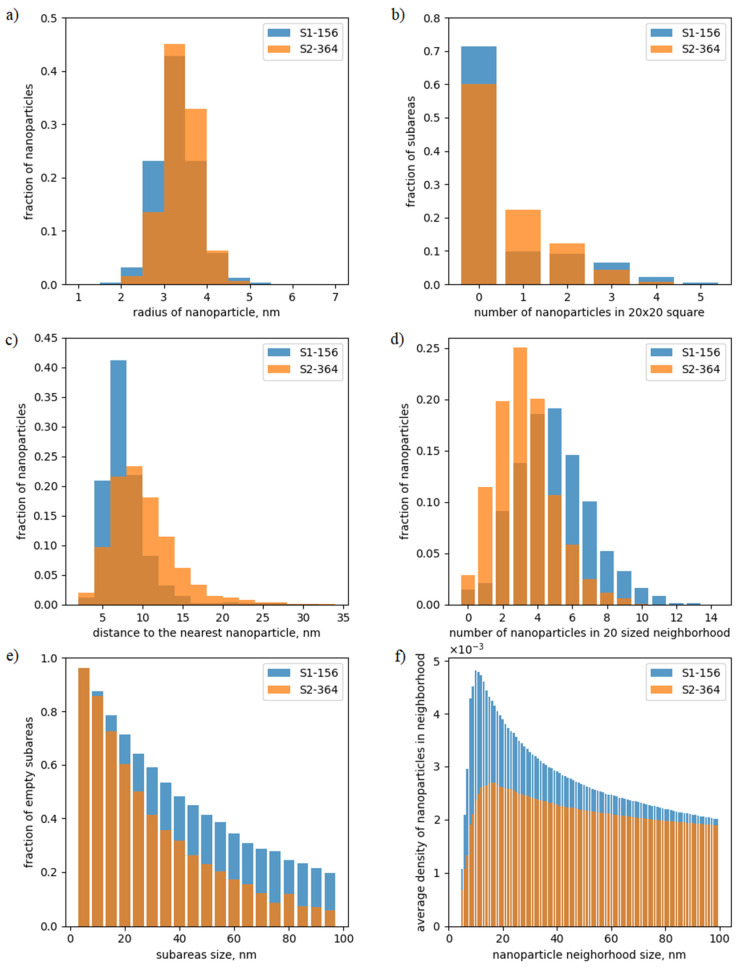
Visual presentation of calculated statistical data for two images from Figure 2 (see the text below for the explanations).

Chart “a” in Figure 3 shows the fraction of nanoparticles of a given radius from the total number of found nanoparticles. Each bar of chart “b” in Figure 3 presents the fraction of 20 × 20 nm subimages that contain the respective number of nanoparticles. The chart “c” in Figure 3 for each distance value to the nearest nanoparticle shows the fraction of nanoparticles for which the respective condition holds true. Each bar of the chart “d” in Figure 3 shows the fraction of nanoparticles with the respective number of nanoparticles in its 20 nm sized neighborhood. Chart “e” in Figure 3 presents the fraction of subimages without nanoparticles from the total number of square subimages of the given size. The chart “f” in Figure 3 shows the average density of nanoparticles inside a circular neighborhood of different radii (in nm). The average density is the number of nanoparticles inside the neighborhood, normalized to its area and averaged over all nanoparticles.

As shown in Figure 3, many proposed statistics are sensitive to the density of nanoparticle locations and the presence of areas without nanoparticles. These data will further allow us to estimate the ordering of the short-range order and to distinguish between types of images.

**Figure 4:**
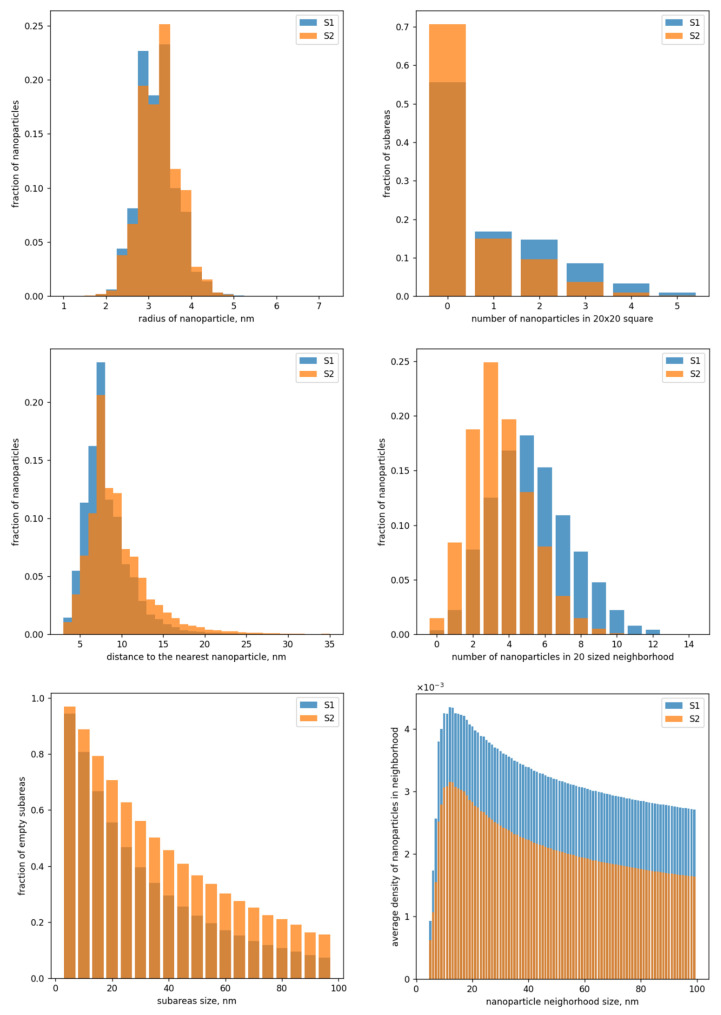
Charts averaged through all images of types S1 (blue) and S2 (orange).

In particular, the presented statistics allow distinguishing quite well between S1 and S2 types by averaging the considered statistics through all images of each of the two types. Figure 4 shows the visualization of the respective averaged statistics. 

**Figure 5:**
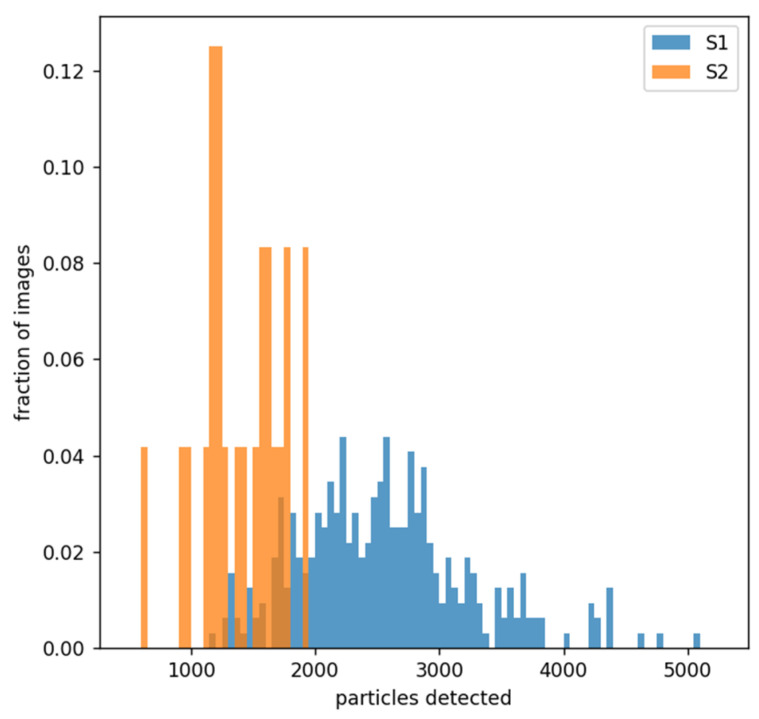
Number of nanoparticles that were detected for S1 (blue) and S2 (orange) images.

In addition to the charts presented in Figure 4, we can plot an additional diagram for all images that shows the number of nanoparticles detected (Figure 5). This diagram shows a trend for S1 images to have more nanoparticles detected than S2 images.

**Figure 6:**
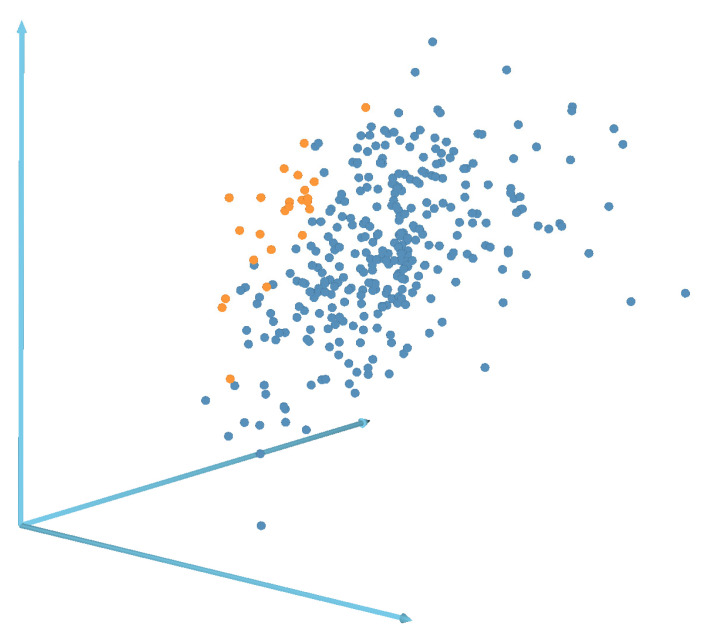
Fast-map three-dimensional projection of the multidimensional feature space. S1 images are drawn in blue, and S2 images are drawn in orange.

For a more accurate analysis, we represent all images as points in the multidimensional feature space. The values of the presented diagrams were taken as features. As a result, each image is characterized by a vector of 177 features. To visualize the relative position of points in the obtained space, they were projected into three-dimensional feature space via a fast-map procedure [32]. The result of the respective visualization is presented in Figure 6. Red points correspond to S1 images, and blue points are S2 images.

As seen from Figure 6, most of the S1 and S2 images form separate and sufficiently concentrated groups of points in the space under consideration, which once again confirms that the proposed statistics make it possible to distinguish images of these types from each other quite well.

It is important to note the chemical nature of samples S1 and S2. They were prepared by precisely the same procedure, so this study shows that small experimental variations can be detected by the described approach.

## 3. Conclusions

As a result, a comprehensive comparison of multiple computer vision approaches for nanoparticle detection was performed. The analysis has shown that the exponential approximation method generally outperforms other methods. However, all methods were shown to be quite sensitive to the hyperparameter choice, requiring the implementation of intelligent approaches for their selection in the future.

The best method was applied to the SEM Pd/C images dataset. Statistical analysis of nanoparticle sizes and their spatial distribution allowed differentiation between two very close materials prepared by the same procedure—one of the key tasks that emerged from experimental studies in the fields of nanoparticle studies and catalysis. 

The performance findings confirm the ability of rapid computer vision algorithms to detect very small changes under experimental conditions. In the future, this will allow fine-tuning of the structure and defectiveness of the surface, thereby changing its physical and chemical properties. The performance of modern CPUs should be enough to extend the analysis into real-time mode.

## 4. Methods

### 4.1. Image Labeling

All images were labeled manually by the expert in the Digimizer image analysis software. The data were then exported into a CSV file and used for the following analysis.

### 4.2. Metric Calculation

To assess the correctness of determining the position and size of the nanoparticle found by the algorithm, we used a method based on the calculation of the Jaccard measure

(1) $$J(S_{gt},S_{dt})=\frac{\left|S_{gt}\cap S_{dt}\right|}{\left|S_{gt}\cup S_{dt}\right|}$$

between circles, the centers of which determine the positions, and the diameters determine the size of the nanoparticle, where $S_{gt}$ is the ground truth circle selected by the expert, and $S_{dt}$ is the circle found by the detection algorithm. The greater the value of this measure, the smaller the difference between the object found by the algorithm and that manually labeled. When the value is equal to one, the found areas completely coincide.

**Figure 7:**
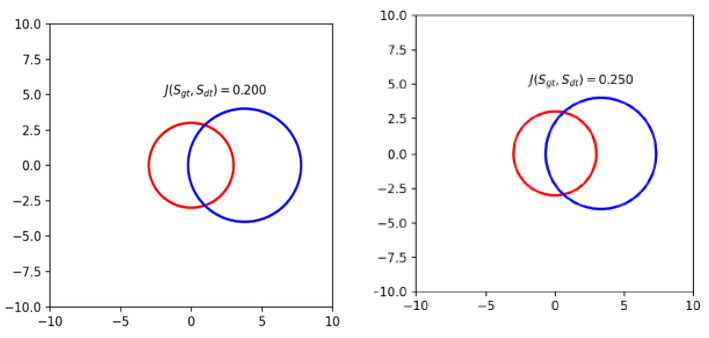
An example of the mutual arrangement of two circles with the Jaccard measure equal to 0.20 and 0.25.

In experiments, the value of the Jaccard measure, at which the corresponding nanoparticle is considered as found by the algorithm, is 0.25. Figure 7 shows an example of the mutual arrangement of two circles with radii of 3 and 4 pixels and an offset of the centers, in which the Jaccard measure is 0.25.

It is worth noting that most algorithms for detecting objects in images deal with rectangular areas of interest (ROIs), and the calculation of the Jacquard measure for two circles was not implemented in standard software packages. However, the nature of the problem being solved, taking into account the specifics of the shape of the detected nanoparticles, requires calculating the measure specifically for circles. The analytical solution for calculating the measure of two circles of radii R and r and located at a distance of d is the following expressions:

(2) $$J=\{\begin{array}{c} \frac{r^{2}}{R^{2}},\;d+r\leq R,\\ \frac{S}{\pi R^{2}+\pi r^{2}-S},\;d-r\leq R,\\ 0,\;d-r>R, \end{array}$$

where $S=AR^{2}-\frac{R^{2}\sin(2A)}{2}+\alpha r^{2}-\frac{r^{2}\sin(2\alpha )}{2}$,

$$A=arc\cos\left(\frac{d^{2}+R^{2}-r^{2}}{2Rd}\right),\;\alpha =arc\cos\left(\frac{d^{2}+r^{2}-R^{2}}{2rd}\right)$$

The quality of the algorithms was evaluated by the accuracy value, which is calculated by the formula

(3) $$accuracy=\frac{TP}{TP+FN+FP}$$

where TP is the number of particles correctly identified by the algorithm, i.e., matching with the expert markup; FN is the number of particles not found by the algorithm but labeled by the expert; and FP is the number of particles found by the algorithm but not confirmed by the expert.

### 4.3. Method Implementation

#### 4.3.1. Classic Methods

The nature of the SEM images allows us to consider the problem of detecting nanoparticles as a classical problem of computer vision, namely blob detection. We included four well-known blob-detection methods in our investigation as baseline methods.

The very straightforward approach is to find the local intensity maxima (LM) of the SEM image and take them as an estimate of the position of nanoparticles if the corresponding intensity value exceeds the given threshold. However, this method does not imply obtaining the particle size. Thus, the size of all particles is assumed to be the same and equal to the average size of the nanoparticle on the image of the given scale.

The Laplacian of Gaussian (LOG) method [33] is the most common blob detector. It is based on the application of the Laplace (second derivative) operator to the result of convolution of the source image with the Gaussian kernel. The scale-normalized version of the Laplacian operator allows us to detect objects of different sizes as local maxima with respect to both space and scale in the three-dimensional discrete scale-space volume. The standard deviations of the Gaussian methods corresponding to the scale at which the maximum is found allowed us to estimate the nanoparticle size.

The difference of Gaussians (DOG) [34] represents a difference between adjacent levels of the image pyramid formed by a sequence of convolutions of the original image with Gaussian kernels with different standard deviations. Then, the ratio between the standard deviations of adjacent levels is equal to approximately 1.6, and the DOG can be considered an efficient approximation of the LOG method.

The fourth method is based on a scale-normalized determinant of the Hessian (DOH) detector [33]. Similar to LOG, nanoparticles are defined from maxima of discrete scale-space volume but formed by the determinant of the Hessian instead of Laplacian. The standard deviation of the Gaussian kernel used for the Hessian matrix with maximum determinant is used for an estimate of the size of the detected nanoparticle.

The application of blob-detection methods is complicated by the presence of overexposed areas on the SEM images. Such areas, as well as background irregularities, must be removed from processing. We propose here to use the greyscale morphology method, namely the top-hat transform [35], to eliminate bright areas that exceed a given size. The top-hat transform is defined as the difference between the original image and its morphological opening. The structuring element of morphological operations plays a critical role in the result of processing. The selection of structuring elements is a subject of intensive studies [36,37].

Fortunately, the visual appearance and known range of possible sizes of nanoparticles simplify the task of structuring element selection and allow the use of a circular element with the size depending on the scale of an SEM image.

In this paper, we used Python implementations of these methods from the scikit-image package skimage.feature.blob_log(), skimage.feature.blob_dog(), and skimage.feature.blob_doh() for LoG, DoG, and DoH, respectively, for nanoparticle detection and skimage.morphology.white_tophat() to preprocess images before applying each of them.

#### 4.3.2. Exponential Approximation (Exp)

The proposed exponential approximation method is based on two assumptions. The first assumption is that the visual representation of each nanoparticle in the image can be modeled quite well using the exponential brightness function with a maximum at the center of the nanoparticle. The second assumption is that the parameters of the approximating functions found for small image fragments will differ for fragments that contain and do not contain a nanoparticle.

The proposed method consists of five stages: (1) preprocessing, (2) selecting small image fragments, (3) making exponential approximation for each image fragment, (4) detecting fragments that contain a nanoparticle, and (5) determining the radius of each particle.

Stage 1. Preprocessing

For the classical methods of blob detection, the top-hat transformation described above was applied to exclude overexposed areas on the SEM images before the main processing.

Stage 2. Selecting small image fragments

The simplest way to select small image fragments is to pass sequentially through all points of the image and cut out fragments of some size fsize from it with a center at the considered points. However, such a straightforward approach leads to multiple unnecessary computations. It is obvious that the lower average brightness of a small image fragment with a center at a certain point corresponds to the lower probability that this point is the nanoparticle center. In this regard, to reduce computations, we introduce an adaptive threshold $Th_{pref}$, which is computed as follows:

$$Th_{pref}=Cf_{pref}mean(LM_{1000})$$

where $mean(LM_{1000})$ is the mean value of the 1000 largest local intensity maxima of an image to be analyzed, and $Cf_{pref}\in (0;1)$ is the coefficient, which is the structural parameter of the method.

For further processing, we select only those points whose average value of brightness in their vicinity exceeds the value of this threshold.

Therefore, the result of this stage is a number of small image fragments.

Stage 3. Exponential approximation of one small image fragment

Let $f=\left[f(x_{i},y_{j}),\;i,j=1,\ldots ,fsize\right]$ be a small image fragment, which has an odd size fsize.

For each fragment, we used the same pixel coordinates $x_{i},y_{j}\in \left\{-\left\lfloor fsize/2\right\rfloor ,\left\lfloor fsize/2\right\rfloor \right\}$ and i,j=1,…,fsize that are relative to the fragment center $\left(x_{\left\lfloor fsize/2\right\rfloor }=0,y_{\left\lfloor fsize/2\right\rfloor }=0\right)$.

An exponential function of the form $F(x,y,a,b)=ae^{-b(x^{2}+y^{2})}$ is used to approximate an image fragment.

The approximation is made in two steps. At the first step for each value of the coefficient b from some finite set $b\in 𝔹\{b_{1},\ldots ,b_{n}\}$, the optimal value a is computed to minimize the root mean square criterion:

(4) $$J(x,y,a,b_{k})=\sqrt{\sum_{i,j}{\left(F(x_{i},y_{j},a,b_{k})-f(x_{i},y_{j})\right)}^{2}},$$

(5) $$a(b_{k})=\underset{a}{\arg\min}J(x,y,a,b_{k}),\;k=1,\ldots ,n$$

In accordance with the least square method, the optimal value of the parameter a can be easily found by the formula

(6) $$a(b_{k})=\frac{\sum_{i,j}f(x_{i},y_{j})e^{-b_{k}{\left(x_{i}+y_{j}\right)}^{2}}}{\sum_{i,j}{\left(e^{-b_{k}{\left(x_{i}+y_{j}\right)}^{2}}\right)}^{2}}$$

In the second step, the parameter values $b_{k}$ and $a(b_{k})$, which lead to the minimum criterion value, are taken as the optimal values of the desired coefficients $b^{opt}=\underset{b_{k}}{\arg\min}J(x,y,a(b_{k}),b_{k}),\;a^{opt}=a(b^{opt}),\;k=1,\ldots ,n$ of the approximating function

(7) $$F(x,y,a,b)=ae^{-b(x^{2}+y^{2})}$$

Stage 4. Detecting fragments that contain a nanoparticle

As a result of exponential approximation for image fragments, we have two matrixes of optimal coefficients $a^{opt}=[a_{i,j}^{opt}]$ and $b^{opt}=[b_{i,j}^{opt}]$, i=1,…,sizeX,j=1,…,sizeY, whose size is equal to the image size to be analyzed.

To detect fragments that contain a nanoparticle, we find local maxima in the matrix $a^{opt}$ and take only those of them whose values exceed the adaptive threshold value

(8) $$Th_{detect}=Cf_{detect}mean(LM(a^{opt}))$$

where $mean(LM(a^{opt}))$ is the average value of all found local maxima in $a^{opt}$, and $Cf_{detect}\in [0;1]$ is the coefficient, which is one more structural parameter of the method.

Stage 5. Determining the radius of a particle

The radius of a particle with center coordinates (i,j) is defined by the respective optimal value $b_{i,j}^{opt}$ as follows:

(9) $$r_{i,j}=\frac{1}{\sqrt{b_{i,j}^{opt}}}$$

#### 4.3.3. Deep Neural Network (DNN) Predictions

The pretrained model was used from the paper. The inference code was implemented in Python with the PyTorch framework [38].

The following procedure was implemented as described previously [39]. Segmentation maps were binarized, then distance transform was applied, centers of nanoparticles were selected by local maxima search algorithm, and implemented in Scikit-learn [40]; finally, the images after distance transform and corresponding local maxima were passed into the watershed algorithm to perform instance segmentation of nanoparticles. 

## Figures and Tables

**Table 1:** Accuracy of methods for the optimal parameter values.

Method	Image				Mean ± SD
	i4	i61	i42	i21	
LoG	75.79	66.40	76.23	**84.62**	75.76 ± 6.45
DoG	**78.42**	72.81	**79.15**	82.43	**78.20 ± 3.46**
DoH	57.93	57.38	62.92	81.70	64.98 ± 9.89
LM	73.90	67.52	69.92	79.46	72.70 ± 4.52
Exp	75.52	**74.77**	77.42	84.30	78.00 ± 3.76
DNN	73.15	69.49	76.13	82.13	75.23 ± 4.63

**Table 2:** Accuracies of nanoparticle detection for parameters estimation based on the leave-one-out procedure.

Method	Images for Finding Parameters/Test Image				Mean ± SD
	i21, i42, i64/i4	i21, i42, i4/i61	i21, i4, i64/i42	i4, i42, i64/i21	
LoG	58.44	64.47	70.52	80.30	68.43 ± 8.07
DoG	64.98	69.06	75.55	70.18	69.94 ± 8.36
DoH	39.08	47.71	48.33	70.82	51.48 ± 11.75
LM	**73.90**	67.52	69.92	73.33	71.16 ± 2.60
Exp	73.75	**72.93**	**75.58**	**83.48**	**76.44 ± 4.18**
DNN	69.88	67.95	76.09	79.59	73.38 ± 4.68

## Data Availability

The data presented in this study are available in the article.
